# Supporting near-peer teaching in general practice: a national survey

**DOI:** 10.1186/s12909-016-0662-9

**Published:** 2016-05-12

**Authors:** Thea F. van de Mortel, Peter L. Silberberg, Christine M. Ahern, Sabrina W. Pit

**Affiliations:** 1 School of Nursing and Midwifery, Griffith University, Parklands Drive, Southport, QLD 4222 Australia; 2 School of Health and Human Sciences, Southern Cross University, Lismore, Australia; 3 North Coast GP Training, Ballina, Australia; 4 University Centre for Rural Health, North Coast, Lismore, Australia

**Keywords:** Vertical integration, Near-peer teaching, General practice, Family medicine, Registrars, Medical students

## Abstract

**Background:**

Training bodies see teaching by junior doctors and vocational trainees in general practice (family medicine) as integral to a doctor’s role. While there is a body of literature on teacher training programs, and on peer and near-peer teaching in hospitals and universities, there has been little examination of near-peer teaching in general practice. Near-peer teaching is teaching to those close to oneself but not at the same level in the training continuum. This study investigated the perceptions of key stakeholders on near-peer teaching in general practice, their current near-peer teaching activities, and methods of recruitment and support.

**Methods:**

A national anonymous online survey was used to obtain data on Australian stakeholders’ perceptions of, and processes related to, near-peer teaching in general practice. Recruitment occurred via electronic invitations sent by training providers and stakeholder associations. Separate questionnaires, which were validated via several cycles of review and piloting, were developed for supervisors and learners. The survey included both fixed response and open response questions.

**Results:**

Responses (*n* = 1,122) were obtained from 269 general practitioner supervisors, 221 general practice registrars, 319 prevocational trainees, and 313 medical students. All stakeholder groups agreed that registrars should teach learners in general practice, and 72 % of registrars, 68 % of prevocational trainees, and 33 % of medical students reported having done some teaching in this setting. Three-quarters of supervisors allowed learners to teach. Having another learner observe their consultations was the most common form of teaching for registrars and prevocational trainees. Eight percent of registrars received some remuneration for teaching. The approach used to determine teaching readiness and quality varied greatly between supervisors.

**Conclusions:**

Near-peer teaching was supported by the majority of stakeholders, but is underutilised and has poor structural support. Guidelines may be required to help supervisors better support learners in this role and manage quality issues related to teaching.

## Background

There is a body of international literature on why medical students, junior doctors and vocational trainees should teach [1–3] and on how to upskill them to teach [4–8]. Studies in university and hospital settings suggest that teaching to peers or near-peers (those that are closer to oneself but not at the same level in the training continuum) improves the learning of the near-peer teacher and does not disadvantage the recipient of the teaching when compared to outcomes of teaching by faculty or standard supervisors [5, 9–13]. Training bodies also see teaching by junior doctors (prevocational trainees) and vocational trainees in general practice (herein referred to as GP registrars (GPR)) as integral to a doctor’s role [14–17]. For the purposes of this study a junior doctor is defined as one that has not embarked on any specialty training, while a vocational trainee is a doctor that is undergoing specialty training, in this case general practice training. Teaching was defined as ‘structured education sessions such as lectures, tutorials, case discussions, journal clubs, ward rounds and wave or parallel consulting with medical students’ [18].

In the Australian setting, an additional driver of the move towards increased near-peer teaching (NPT) by learners in general practice is that Australian GP supervisors (GPS) often work on a fee for service basis, so taking time out to supervise trainees can impact on their income. The sector has also experienced a steep increase in learners requiring placements and training in general practice [19]. Similar training capacity constraint issues are reported in the United Kingdom (UK) [20, 21].

Dick et al. [22] has proposed that learning in the general practice setting should be multi-directional, ie. that GPS, GPR, prevocational trainees (PT) and medical students (MS) should all teach and learn from one another thus reducing the burden on the supervisor and potentially increasing training capacity. However, there is limited research in the general practice setting to support policy settings on near-peer teaching. Between 35 % and 62 % [23, 24] of GPR report doing some teaching in Australia and the UK. Of the Australian GPR who report teaching, 82 % taught medical students, 34 % taught other registrars, and 20 % taught PT [24]. There are no data on teaching by PT and MS in general practice. Nine percent of Australian [25], and 41 % of British GPR [23] reported receiving teacher training, however these studies are limited by small samples.

Various Australian and British studies have examined aspects of NPT in general practice, primarily barriers and facilitators and/or benefits and disadvantages of near-peer teaching [22, 26, 27], and motivation to teach [28]. Key findings to date are that many participants see benefits to learners teaching in general practice including increased collegiality and multi-directional learning in the practice, improved learning for the near-peer teacher, and a potential reduction in supervisor burden. Some suggested barriers include lack of space and teacher training, impacts on income (for senior registrars who get a proportion of their income from billings), and concerns that near-peer teaching may reduce the quality of the learning experience for teaching recipients. However, these studies have largely focused on the perceptions and experiences of GPR and GPS [22, 26], and have been characterised by study designs that limit the generalizability of the findings, for example, small samples and qualitative or case study design [22, 27, 28], sampling localised to one training region [26–28], or use of a partially validated survey instrument [26]. To our knowledge there is no literature from the general practice setting on:

1. how learners are selected for, and supported in, a peer teaching or NPT role,
2. how the quality of the teaching is determined by the supervisor,
3. the degree to which supervisors allow learners to take on teaching roles,
4. the degree to which learners at the various training levels want to teach , and
5. how well learners feel supported to teach.

This study investigated the perceptions of key stakeholders on near-peer teaching in general practice, their current near-peer teaching activities, and methods of recruitment and support, to inform future NPT medical education practices.

## Methods

### Study design

A cross sectional design was utilised. The views of Australian GPS, GPR, PT, and MS on NPT in general practice were obtained via a national anonymous online survey.

### Sample and recruitment

Recruitment occurred via invitations in electronic newsletters and emails disseminated by training providers and national stakeholder associations. A sample of 346 GPS, 333 GPR and 123 PT was sought to obtain a confidence level of 95 %, and a confidence interval of 5 [18], based on 2010 data [29] on numbers of learners and supervisors in general practice. The MS sample was estimated at 200. Participants could enter a draw for an iPad. Completion of the anonymous online survey was considered to be consent.

### Instrument development and validation

Questionnaire items were developed based on a literature review, previous qualitative data obtained by the researchers, and advice from a panel of stakeholders. Supervisors and learners completed separate instruments. The questionnaires (see link)^1^ were validated via several cycles of review and piloting [18]. The survey items specifically investigated:

Via non-scale selected response questions:

- Stakeholders’ perceptions of who should teach in general practice.
- The percentage of:
  - ○ respondents who were located in single-level-learner (SLL) or multi-level-learner (MLL) practices (an example of the latter is a practice with a GPR and MS).
  - ○ learners undertaking clinical supervision of juniors in general practice, eg. parallel consulting.
  - ○ learners delivering other types of teaching to peers or near-peers.
  - ○ GP supervisors who allowed learners to teach, and what those teaching roles were.
- The type of training participants received for teaching roles.
- How learners were supported in their teaching roles.

Via 5-point Likert scales:

- Stakeholders’ perceptions of:
  - ○ who should teach in general practice (Teaching Roles Scale).
  - ○ the barriers to, and facilitators of, ‘learners as teachers’ in general practice and how they ranked them in importance.
  - ○ how well learners were supported in teaching roles (Teaching Support Scale).
- Stakeholders’ beliefs about near-peer teaching (Near-peer Teaching Scale).

Via open response questions:

- Stakeholders’ views on how to improve the quality of clinical supervision in general practice and increase training capacity.

Additional opportunities for comments were provided throughout the survey.

### Ethics approval

Southern Cross University’s Human Research Ethics Committee provided ethics approval.

### Data analysis

Descriptive statistics were calculated (mean ($\overline{\chi}$) values are followed by the standard deviation). Chi^2^ tests were used to investigate differences in proportions, while differences between groups were examined using a t-test (with a Bonferroni correction to reduce Type 2 errors) for continuous variables when two groups were examined, and Analysis of Variance when more than two groups were examined. Cronbach’s alpha was used to determine scale reliability. Statistical analyses were conducted using SAS version 9.2 (SAS Institute, Cary, NC, USA). Thematic analysis was conducted on qualitative comments using the method described by Braun and Clarke [30].

## Results

### Demographics

A total of 1,122 stakeholders completed the survey: 269 GPS, 221 GPR, 319 PT, and 313 MS (Table 1) [18]. Respondents represented approximately 14 % of GPS and 7 % of GPRs who were nationally registered [18]. Our sample compared well to the demographics of these groups nationally. For example, the proportion of female supervisors was identical to the national figure and 48 % of supervisors were aged 50–59 years (compared to a national average of 51) [31]. Similarly 68 % of registrars were female compared to 64 % nationally, and 48 % were aged 30–39 years (the average age nationally is 35) [32]. Additionally the breakdown of registrars in urban, rural and remote regions was identical to the national figures [33]. Eighty-nine percent of the medical students were aged less than 30 compared to 84 % nationally, although we had a higher response rate from females (71 % vs 55 % nationally) [34].

**Table 1:** Sample demographics (*n* = 1,122)

Group (*n*)	Stage (%)	MLL (%)	Age	Gender	Location
GPS 269	Supervisor	88 %	<50 36 %	F 41 %	Urban 39 %
	<5 yrs 23 %		50–59 48 %		Rural 59 %
	5–10 yrs 22 %		≥60 16 %		Remote 3 %
	≥10 yrs 54 %				
GPR 221	GPT1 34 %	85.5 %	<30 32 %	F 68 %	Urban 44 %
	GPT2 13 %		30–39 48 %		Rural 52 %
	GPT3 29 %		≥40 20 %		Remote 4 %
	GPT4/ES 24 %				
PT 319	PGY1 31 %	80.8 %	<30 72 %	F 66 %	Urban 38 %
	PGY2 29 %		30–39 22 %		Rural 51 %
	≥PGY3 40 %		≥40 6 %		Remote11%
MS 313	Years 1–2 17 %	60.3 %	<30 89 %	F 71 %	Urban 53 %
	Years 3–4 55 %		30–39 8 %		Rural 43 %
	Years 5–6 28 %		≥40-49 3 %		Remote 4 %

Adapted and reproduced with permission from The Royal Australian College of General Practitioners from: van de Mortel T, Silberberg P, Ahern C, Pit S. Stakeholders’ views of shared learning models in general practice: a national survey. Aust Fam Physician 2014;43(9):633–38

GPT1 = term 1; GPT2 = term 2; GPT3 = term 3; GPT4 = Extension of time; ES = Extended skills term; PGY1 = postgraduate year 1; PGY2 = postgraduate year 2; PGY3 = postgraduate term 3; MLL = Practice with multiple levels of learner present

### Scale reliability

The Near-Peer Teaching and Teaching Roles Scales had adequate to good reliability (0.77-0.82 and 0.77-0.87, respectively), while the Teaching Support Scale had adequate to good reliability (0.72-.86) in all groups except medical students (0.62).

### Who should teach in general practice?

There was agreement from supervisors that registrars, practice nurses and practice managers should have a role teaching learners (Table 2). Supervisors in MLL practices were more likely to agree that registrars (*p* = 0.0003), PT (*p* = 0.003) and MS (*p* = 0.0014) should teach, compared to those from SLL practices. Learners also agreed that registrars and practice nurses should teach, but were more equivocal about teaching roles for other groups.

**Table 2:** Stakeholders’ beliefs about who should have a role teaching learners in general practice ($\overline{\chi}$ ± s.d.)

Statement	GPS *N* = 269*	GPR *N* = 220	PT *N* = 310	MS *N* = 309
GP registrars	4.3 (0.81)	4.0 (0.84)	4.2 (0.71)	4.4 (0.65)
Prevocational trainees	3.5 (1.05)	3.2 (1.06)	3.7 (0.89)	3.8 (0.88)
Medical students	3.2 (1.15)	2.9 (1.12)	3.1 (1.03)	3.3 (1.02)
Practice nurses	4.3 (0.68)	4.0 (0.68)	4.0 (0.74)	4.0 (0.76)
Practice managers	4.1 (0.81)	3.7 (0.91)	3.7 (0.86)	3.6 (0.93)

*Some missing data. 1 = strongly disagree; 2 = disagree; 3 = not sure; 4 = agree; 5 = strongly agree.

Supervisors who already allowed some NPT were significantly more likely to agree that registrars (*P* = 0.002), PT (*p* = 0.005), MS *p* = 0.032), practice nurses (*p* < 0.001), and practice managers (*p* = 0.03) should have a role teaching learners. The most frequent comment (52.5 %) by supervisors who chose to comment on teaching roles was that allied health professionals (including pharmacists, physiotherapists, dieticians, Aboriginal Health Workers, diabetes educators, psychologists, and podiatrists) and other practice staff such as receptionists should also have teaching roles where appropriate. Conversely, 17.5 % of supervisors indicated disagreement with learners teaching due to lack of time and/or training to teach, and lack of assessment of the competency of learners to teach.

There were no statistically significant differences in beliefs about who should teach in general practice between learners in SLL and MLL practices, or between registrars who delivered NPT and those who did not. However, registrars who experienced NPT agreed more strongly that PT should teach ($\overline{\chi}$ 3.4 (±1.02) vs 3.1 (±1.01), *p* = 0.03). Additionally, PT who had conducted NPT had stronger beliefs that the following groups should have a teaching role compared to PT who had not conducted NPT: GPRs ($\overline{\chi}$ 4.3 (±0.85) vs 4.0 (±0.87), *p* = 0.0005), and PTs ($\overline{\chi}$ 3.8 (±0.85) vs 3.5 (±0.87), *p* = 0.0059). Medical students in MLL practices had stronger beliefs that MS should have a teaching role compared to those in SLL practices ($\overline{\chi}$ 3.5 (±1.00) vs 3.0 (±1.10), *p* < 0.0001).

### How many have teacher training?

The percentage of supervisors, registrars, PT, and MS who had some teacher training was 77 %, 65 %, 71 % and 65 % respectively. For supervisors *formal training* via supervisor workshops provided by the regional training provider (RTP) of GP training (*n* = 144) or the GP college (*n* = 12) was the most common type of training, while a small number (*n* = 22) had formal teaching qualifications. Teacher training for learners was most commonly via *informal* teaching tips from their supervisor (Table 3).

**Table 3:** Type of teacher training received by learners (%) (MLL only)

Statement	GPR *N* = 188	PT *N* = 249	MS *N* = 182
None	35 %	29 %	35 %
Teaching tips from supervisors	38 %	46 %	49 %
Teaching workshop provided by RTP	34 %	16 %	3 %
Teaching workshop provided by hospital	24 %	24 %	9 %
Formal teaching qualification	7 %	5 %	3 %

*RTP* regional training provider, *MLL* multi-level learners, *GPR* general practice registrar, *PT* prevocational trainee, *MS* medical student

### What teaching do learners do in general practice?

Twenty-four percent (55/226) of MLL supervisors reported that learners did not do any clinical supervision/teaching in their practice. Table 4 displays types of teaching by learners.

**Table 4:** The types of teaching reported by learners in general practice (%)

Statement	GPS *N* = 226	GPR *N* = 188	PT *N* = 249	MS *N* = 182
None	24 %	28 %	32 %	67 %
Registrar delivers an education sessions to one or more learners	54 %	41 %	-	-
PT delivers an education session to one or more learners	12 %	-	30 %	-
MS delivers an education session to one or more learners	25 %	-	-	16 %
MS develops resources for others to present	10 %	-	-	10 %
MS observes registrar or PT patient consultations	38 %	51 %	56 %	-
Registrar parallel consults with MS	31 %	26 %	-	-
Registrar parallel consults with PT	8 %	9 %	-	-
PT parallel consults with MS	4 %	-	23 %	-

*GPS* general practitioner supervisor, *GPR* general practice registrar, *PT* prevocational trainee, *MS* medical student

### Who has been the recipient of NPT?

Overall, 53 % of GPR, and 71 % of PT and MS, reported having been taught by another learner in general practice, and 34 % of GPR, 43 % of PT and 34 % of MS reported that other learners were teaching in their current practice. Parallel consulting was the most common form of teaching received from other near-peer teachers.

### How are learners selected for and supported in teaching roles?

Learners most commonly are invited or volunteer to teach (Table 5). Overall, the less experienced the learner, the more supported the learners felt in teaching roles (Tables 6 and 7).

**Table 5:** Method of recruitment of learners to a teaching role in general practice (%)

Statement	GPS *N* = 226	GPR *N* = 121	PT *N* = 129	MS *N* = 91
They volunteer	19 %	40 %	54 %	19 %
Invited to teach	54 %	45 %	46 %	19 %
Required to teach by university or RTP	5 %	3 %	1 %	10 %
Expected as part of their role in the practice	28 %	35 %	27 %	10 %
Other	6 %	-	-	-

*GPS* general practitioner supervisor, *GPR* general practice registrar, *PT* prevocational trainee, *MS* medical student, *RTP* regional training provider

**Table 6:** Teaching support for learner-teachers in general practice ($\overline{\chi}$ ± s.d.)

Statement	GPS* N = 161	GPR *N* = 121	PT *N* = 126	MS *N* = 38
Are given constructive feedback on their teaching from GPs in the practice	2.7(1.0)	1.6 (0.76)	2.0(0.90)	2.4(0.75)
Are given feedback on their teaching from other learners	2.6(1.1)	2.0 (0.77)	2.2(0.83)	2.5(0.73)
Are given teaching tips prior to teaching	2.4(1.1)	1.5 (0.62)	1.6(0.81)	2.0(0.90)
Felt supported in the teaching role	-	2.2 (0.88)	2.5(0.78)	2.8(0.77)
Have their teaching resources vetted by a GP prior to delivery	2.1(1.1)	1.3(0.61)	1.6(0.75)	2.0(0.96)

*GPS were asked whether learners are given that type of teaching support. 1 = never; 2 = sometimes; 3 = mostly; 4 = always. *GPS* general practitioner supervisor, *GPR* general practice registrar, *PT* prevocational trainee, *MS* medical student

**Table 7:** Proportion of participants that reported never receiving (or giving*) the following types of teaching support to learner-teachers (%)

Statement	GPS* N = 161	GPR *N* = 116	PT *N* = 126	MS *N* = 38
Are given constructive feedback on their teaching from GPs in the practice	9 %	53 %	36 %	8 %
Are given feedback on their teaching from other learners	13 %	26 %	21 %	5 %
Are given teaching tips prior to teaching	20 %	60 %	56 %	30 %
Felt supported in the teaching role	-	25 %	10 %	3 %
Have their teaching resources vetted by a GP prior to delivery	35 %	73 %	57 %	41 %

*GP* general practitioner, *GPS* general practitioner supervisor, *GPR* general practice registrar, *PT* prevocational trainee, *MS* medical student, *RTP* regional training provider

### Payment for learners

The majority (62 %) of supervisors and registrars reported that registrars were not remunerated for teaching, although 17 % of supervisors reported paying a Practice Incentive Payment (PIP) to registrars for teaching medical students and 10 % paid part of the PIP (Table 8).

**Table 8:** Type of payment received by (or provided to*) registrars who teach (%)

Statement	GPS* N = 129	GPR *N* = 117
Registrar is on salary	-	30 %
No remuneration	62 %	62 %
Whole PIP for parallel consulting with MS	17 %	2 %
Part PIP for parallel consulting with MS	10 %	5 %
Payment for delivery of an education session to others in the practice	11 %	1 %

*PIP* practice incentive payment’, *GPS* general practitioner supervisor, *GPR* general practice registrar

### Assessment of teaching readiness and quality

Eighty-two percent (132/161) of supervisors who allowed NPT reported assessing the near-peer teachers’ readiness to teach and 50 % (80/161) reported assessing NPT quality. Common assessment methods are reported in Table 9. Many supervisors used a combination of strategies. Some supervisors reported that they relied on ‘gut feeling’ or personal judgement of the learner’s capabilities, and considered the learner’s personality in addition to other factors.

**Table 9:** Assessment of teaching readiness and quality

Teaching readiness of learners	
1. Direct observation of the near-peer teaching (*n* = 36). In some cases supervisors would also review the learner-teacher’s slides/resources, or provide teaching tips, feedback, and demonstrations of teaching.	
2. Assessment of the learner’s clinical competency and knowledge (*n* = 30) as a marker for ability to supervise and teach juniors to ensure that correct information was being passed on, and that safety was maintained.	
3. Feedback from the recipients of the teaching (*n* = 17).	
4. Assessment of the learner’s enthusiasm for and willingness to teach (*n* = 14), confidence to teach (*n* = 9) or communication skills (*n* = 4).	
5. Consideration of prior teaching experience (*n* = 4).	
Teaching quality	
1. Direct observation of the teaching/supervision, i.e. sitting in on the session (*n* = 40).	
2. Feedback from the recipients of the teaching (*n* = 38).	
3. Discussion with/debriefing of the learner-teacher. This ranged from asking the learner-teacher how it went, to more in depth attempts to engender reflection in the learner-teacher, such as discussing effective and tricky aspects of teaching/supervision, and asking learners to reflect on how they could improve their teaching (*n* = 13).	
4. Questioning to determine achievement of learning in the recipients of the teaching/supervision (*n* = 4).	

### Beliefs about near-peer teaching (NPT)

Supervisors generally had positive beliefs about NPT (Table 10), although they were unsure about any financial benefit to having learners teach. Supervisors were most positive about the benefits to the learner-teacher and to their own learning from NPT. Learners also had fairly positive beliefs about NPT (Table 11), although their beliefs about the benefits to the learner-teacher were more positive than their beliefs about the benefits to the recipients of that teaching.

**Table 10:** Supervisors’ beliefs about near-peer teaching ($\overline{\chi}$ ± s.d.)

Statement	GPS *N* = 164
Learners teaching can reduce time pressures on supervisors	3.9 (0.86)
Teaching by learner-teachers is generally less effective for the other learners than teaching by supervisors	2.5 (0.86)
Learner-teachers improve their own learning through teaching others	4.4 (0.55)
Getting learners to teach is a form of succession planning	4.2 (0.65)
Having learners teach creates capacity to take on more learners	3.9 (0.94)
Learners in the practice enjoy being taught by another learner	3.9 (0.74)
Supervisors can learn from presentations delivered by learners in the practice	4.5 (0.55)
Having learners teach is financially beneficial to the practice	3.1 (1.00)
Learners in general practice often have prior skills and knowledge to contribute	4.3 (0.74)
GP registrars often have prior experience teaching juniors	3.8 (0.86)

*GPS* General practitioner supervisor, 1 = strongly disagree; 2 = disagree; 3 = not sure; 4 = agree; 5 = strongly agree

**Table 11:** Learner’s beliefs about near-peer teaching ($\overline{\chi}$ ± s.d)

Statement	GPR	PT	MS
Completed by those who received NPT	*N* = 94	*N* = 148	*N* = 114
Teaching by learner-teachers is generally less* effective for me than teaching by supervisors	3.4(1.05)	3.3(0.95)	3.3(0.99)
I enjoy being taught by another learner	3.8(0.71)	3.9(0.58)	4.0(0.57)
Having learners teach creates capacity to take on more learners	3.5(0.79)	3.7(0.73)	3.8(0.79)
Registrars often have a better understanding of the learning needs of medical students than GPs	3.6(0.92)	3.6(0.89)	3.9(0.91)
Learners in general practice often have prior skills and knowledge to contribute	4.0(0.66)	4.0(0.58)	4.0(0.75)
GP registrars often have prior experience teaching juniors	3.8(0.72)	3.7(0.74)	3.9(0.76)
Completed by those who conducted NPT	*N* = 117	*N* = 128	*N* = 38
Learner-teachers improve their own clinical skills through teaching	4.3(0.71)	4.3(0.55)	4.3(0.84)
Learner-teachers improve their own communication skills through teaching	4.3(0.66)	4.3(0.62)	4.5(0.69)
Learner-teachers improve their own knowledge through teaching	4.4(0.62)	4.4(0.53)	4.5(0.69)
Teaching is a way of giving back to the profession	4.3(0.62)	4.4(0.53)	4.5(0.60)
Teaching opens up an additional career pathway	4.1(0.80)	4.1(0.75)	4.0(0.84)

*Reversed during data analyses. GPR general practice registrar, *PT* prevocational trainee, *MS* medical student,1 = strongly disagree; 2 = disagree; 3 = not sure; 4 = agree; 5 = strongly agree

Supervisors perceived the two most important facilitators to be “learners who are enthusiastic about teaching” and “knowing the capabilities of the learner-teacher” (Table 12). Learners thought that enthusiasm by the near-peer teacher, access to clinical resources and mentoring were important facilitators of NPT. Perceptions of the importance of barriers to NPT varied between groups (Table 13). MLL supervisors perceived the two most important barriers to be “concerns about overloading learner-teachers” and “concerns about the quality of the teaching provided by learner-teachers”. SLL supervisors generally rated perceived barriers to NPT as more important than MLL supervisors. Registrars who had conducted NPT were more likely to see *“Loss of income to registrars”* ($\overline{\chi}$ 2.7 (0.92) vs 2.3 (0.92), *p* = 0.0095) as an important barrier compared to those who had not done any NPT.

**Table 12:** Facilitators of near-peer teaching in general practice ($\overline{\chi}$ ± s.d.)

Statement	GPS *N* = 217 MLLs	GPS *N* = 29 SLLs	GPR *N* = 195	PT *N* = 246	MS *N* = 157
Teacher training for the learner-teacher	3.1(0.77)	3.1(0.87)	3.1(0.81)	2.9(0.78)	3.0(0.86)
Learners who are enthusiastic about teaching	3.6(0.58)	3.7(0.55)	3.4(0.64)	3.3(0.67)	3.6(0.61)
Access for learner-teachers to educational resources, eg. models, whiteboard	2.9(0.90)	3.5(0.68)	2.8(0.84)	2.9(0.81)	2.9(0.81)
Access for learner-teachers to clinical resources, eg. guidelines, online tools	3.2(0.79)	3.5(0.69)	3.4(0.65)	3.2(0.70)	3.2(0.72)
Access for learner-teachers to software such as PowerPoint and training in skills to use it	2.6(1.00)	2.9(0.97)	2.5(0.92)	2.6(0.89)	2.6(0.90)
Knowing the capabilities of the learner-teacher	3.4(0.64)	3.4(0.69)	3.3(0.67)	3.1(0.68)	3.3(0.71)
Efficient administrative support	3.2(0.77)	3.4(0.73)	3.0(0.74)	2.9(0.72)	-
Paying registrars to teach	-	-	3.0(0.94)	2.9(0.82)	2.8(0.85)
Mentoring for learner-teachers	-	-	3.2(0.79)	3.0(0.71)	3.2(0.72)

*GPS* general practitioner supervisor, *GPR* general practice registrar, *PT* prevocational trainee, *MS* medical student, 1 = unimportant; 2 = mildly important; 3 = important; 4 = very important

**Table 13:** Barriers to near-peer teaching in general practice ($\overline{\chi}$ ± s.d.)

Statement	GPS *N* = 217 MLL	GPS *N* = 29 SLL	GPR *N* = 194	PT *N* = 246	MS *N* = 262
Loss of income to GP supervisors	2.3 (1.10)	2.9(1.1)	2.6(0.94)	2.6(0.87)	2.5(0.92)
Loss of income to registrars	2.4 (0.95)	2.8(0.98)	2.6(0.95)	2.6(0.88)	2.5(0.90)
Learners' short placements	2.3 (0.97)	2.3(0.97)	2.2(0.91)	2.4(0.88)	2.5(0.84)
Learners who lack confidence in their teaching skills	-	3.0(0.82)	2.8(0.85)	2.8(0.82)	3.1(0.80)
Fears about patient safety when registrars parallel consult with medical students	2.5 (0.92)	2.7(0.85)	2.5(0.93)	2.3(0.88)	2.4(0.91)
Concerns about overloading learner-teachers	2.8 (0.81)	3.1(0.75)	2.9(0.87)	2.7(0.78)	2.8(0.82)
Concerns about the quality of teaching provided by learner-teachers	2.7 (0.78)	3.2(0.63)	2.9(0.80)	2.8(0.82)	2.9(0.83)

*GPS* general practitioner supervisor, *GPR* general practice registrar, *PT* prevocational trainee, *MS* medical student,1 = unimportant; 2 = mildly important; 3 = important; 4 = very important

#### What would improve general practice training quality or capacity?

Suggestions to improve training quality and capacity in relation to NPT are displayed in Table 14.

**Table 14:** Suggestions by key stakeholders to improve training quality and capacity

Supervisors	
1. Have better career paths for potential teachers that are appropriately remunerated.	
2. Pay registrars a salary to reduce reliance on fee for service and impacts of NPT on registrars’ remuneration.	
3. Better support from RTPs:	
a. Make teaching training for GPR a core component of RTP teaching	
b. Provide training to GP supervisors on the concept of the ‘learning organisation’.	
Registrars	
1. ‘*Good doctor does not always make good teacher*.’ More training on teaching is required for doctors who teach.	
2. Support those registrars that want to teach. ‘*I put my hand up to teach and got queer looks and no form of support whatsoever, so now I teach privately*.’	
3. The fee for service structure of general practice presents a barrier to teaching by registrars.	
4. Stratify the teaching, for example, the supervisor primarily teaches junior registrars; senior registrars teach the PTs, and junior registrars and practice nurses teach the MS.	
Junior doctors	
1. Registrars should be given the opportunity, training and resources to teach, but teaching should not be compulsory.	
Students	
1. Support for keen GPRs to teach is vital as they are an excellent (and approachable) resource for medical students.	
2. Educate practices about what medical students have to offer the practice. They have often covered the latest research at university, and are often postgraduates with a great deal of life experience, and as such can also contribute to knowledge in the practice, helping the supervisor and practice to keep up to date with the latest approaches.	

## Discussion

The more exposed key stakeholders are to new models of teaching, the more receptive they are to these. Supervisors located in MLL practices were more likely to agree that registrars, junior doctors and MSs should be teaching when compared to those from SLL practices. Additionally, NPT experience reduced perceptions of the importance of barriers to NPT. This suggests that if NPT models are to be successfully implemented on a widespread basis, innovative ways of exposing GP supervisors and learners to NPT need to be found. Van de Mortel et al. [30] found in a small pilot study that GPs and learners who learnt and taught about adolescent health in a vertically integrated model developed more positive views towards learners teaching in general practice. The intervention showcased some of the skills that juniors were able to bring, which made both supervisors and learners more positive about what learners could offer.

Utilising practice nurses, practice managers and allied health professionals to do some teaching was considered by supervisors to be a useful strategy to increase training capacity but they were less likely to see a teaching role for junior doctors and MS. About three-quarters of supervisors allowed some form of NPT in their practice, and around 70 % of registrars and PT reported doing some form of NPT while about one third of medical students did. About half of the registrars and PT had a medical student observe their consultations, while about one quarter parallel consulted with medical students. So there appears to already be some acceptance of the idea that some registrars and to a slightly lesser extent, prevocational trainees can clinically supervise medical students. This is unsurprising given the amount of supervision these groups do in the hospital setting. Teaching by medical students was much less common and most likely to take the form of group presentations and preparation of teaching materials for supervisors or senior learners. Only 12 % of students had participated in a teacher training workshop compared to 40 % of junior doctors and 56 % of registrars, and two third of MS did no teaching at all. Medical schools could consider including teaching as a component in the curricula to increase early exposure to teaching and increase students’ confidence to become teachers early in their career, and thereby increasing future training capacity. Some schools are already either doing so or are advocating for this to occur [31].

Supervisors’ views on NPT were generally positive, however, some felt that juniors lacked knowledge, skills and wisdom and should therefore not teach. Some supervisors also believed that learning was one-directional, ie. the GP is the teacher. There were concerns about dissemination of incorrect information by near-peer teachers. Indeed, one supervisor had been at risk of legal action due to a mistake made when a learner was supervising another learner. NPT practice implementation should coincide with developing compliance or quality assurance systems to ensure legal and professional rules and regulations are safeguarded. A safe way of reducing pressure on the supervisor involves the medical student preparing slides for the supervisor to present at an education session. The medical student learns about the topic through researching it, while the GPS reduces teaching preparation time through the medical student sourcing images, and resources on their behalf. Currently, only 10 % of medical students reported providing this kind of teaching support, thus there is room to increase teaching capacity by medical students.

Conversely, some supervisors saw the GP practice as a learning organisation for all its members (including the supervisor), that learning was multi-directional, and that juniors could bring new knowledge and also skills to the practice. For example, registrars had often done some other postgraduate training (eg. paediatrics, surgery). Given that many programs of medicine are now postgraduate entry, it is also becoming more common for medical students to come into medicine with graduate qualifications in other health fields such as pharmacology and physiotherapy. This theme emerged strongly in a previous small qualitative study of NPT [27]. A UK study demonstrated that a junior doctor led NPT prescribing program increased the self-reported confidence, knowledge and prescribing skills of medical students [32], which demonstrates the skills that juniors can bring to medical education. NPT systems implementation should include a component that investigates the learners’ knowledge in terms of postgraduate training, previous health-related careers, and basic sciences. This information should be used to develop local NPT training sessions.

Knowing the capabilities of the learner-teacher was considered to be one of the most important facilitators of NPT by supervisors, along with learner enthusiasm, efficient administrative support and teacher training. Learners also saw enthusiasm for teaching, access to clinical resources and teacher training as important along with mentoring for learner-teachers. Successful learner-teacher programs have been piloted as part of a curriculum. For example, in a US medical school, the Medical Education Pathway (MEP) teaches MS how to teach thereby addressing local training needs and preparing students for future teaching roles. The program is embedded within the curriculum as an elective. MSs receive mentoring, assessment, and formative feedback on lecture delivery and leadership of various small-group formats [33].

Supervisors did feel overall that NPT could increase clinical supervision capacity, although they were equivocal about whether NPT could be financially beneficial to the practice. In Australia, the majority of supervisors and registrars work in private practices so teaching students diminishes monetary income. This seems to particularly problematic in rural areas as a recent study found that teaching in rural practice was significantly less cost-effective than in urban areas [34]. Interestingly, Laurence et al. [35] demonstrated that having the registrar carry out clinical supervision of juniors increased financial returns to the practice, which suggests that practices may benefit from exposure to cost-benefit analyses to show the clear benefits that can accrue to practices. Loss of income to registrars who are learner-teachers was not rated highly as a barrier by learners or supervisors, but this is partly explained by the fact that one-third of registrars were salaried, or received some form of payment when parallel consulting with MSs. One suggestion was to reduce the reliance of senior registrars on fee for service and have them paid a salary via a central training body.

The big gap in current processes is in determining when a learner is ready to take on a teaching/supervision role, and how to prepare them for and support them in that role. Attitudes and practices reported by supervisors varied from:

1. We should have a structured way to assess this but are not currently doing so.
2. We let them teach and see how they go.
3. Give them tips on teaching prior to taking on a teaching role, and support them through the process by giving them feedback to help them improve their teaching skills and confidence.
4. Assess the learners’ teaching skills and competence prior to getting them to teach a wider group by first having them deliver an education session to the supervisor.

Aside from actually observing a teaching session, supervisors reported that signs that the learner was ready to teach included their knowledge and clinical skill, level of enthusiasm, communication skills and confidence, and also ability to recognise their own limitations and seek help if required.

These varied approaches were reflected by learners reporting that on average they sometimes felt supported in the teaching role. The lower the level of the learner, the more likely they were to report higher levels of support for teaching from supervisors and other learners, indicating recognition that more junior learners required more help to teach. In terms of individual types of teaching support, registrars reported rarely receiving teaching tips, or constructive feedback from their supervisors about their teaching, or having someone vet their teaching resources. A small number of supervisors indicated that they tried to encourage reflection on teaching. If NPT is to contribute to increased training capacity then supporting learner-teachers in the role, and helping them to develop reflective teaching practice is vital as reflective practice in teaching can improve teaching quality and the confidence of teachers [36, 37]. Mentoring for learner-teachers was considered an important facilitator of NPT by learners. In this sample of learners, the most common form of teacher training was in the form of tips from supervisors. There was little more structured training, although some RTPs do teacher training sessions with their registrars. Some form of teacher training was recognised by learners to be a moderately important facilitator of NPT.

Some stakeholders expressed the opinion that some are born teachers, and others shouldn’t teach because they are not very good at it, are poor communicators, lack knowledge, or are disinterested. However, an important part of being a diagnostician, and effectively managing illness prevention and treatment in primary care involves engaging, educating and motivating patients to improve their health and outcomes. If the learner does not have these skills then perhaps they of all people need support to gain these skills.

### Strengths and weaknesses of the study

This study has multiple strengths. Firstly, this is the first study that provides national Australian data on key stakeholders’ views on NPT experiences across a wide range of general practices including rural and urban practices, and those that are currently utilising NPT to those who are not. In particular, medical students and junior doctors have not previously had a voice in the literature on their views on NPT except for several small qualitative studies conducted in localised areas [22, 25–27]. Secondly, the questionnaire was also developed with input from, and testing on the key groups of stakeholders. Survey validity and reliability were established and the design of the survey not only collected quantitative data on the variables of interest, but allowed opportunities for respondents to clarify their comments or to add additional comments and suggestions to provide a comprehensive examination of these issues. Thirdly, the study can be translated into practice to increase teaching capacity by following the recommendations generated by this research (Table 15).

**Table 15:** Recommendations for clinicians, policymakers and educators

Embed NPT in curricula and professional development activities throughout medical career	
1. That RTPs and universities ensure that NPT skills are considered a core competency.	
2. That RTPs and universities provide some NPT training as a matter of course.	
3. That RTPs and universities make some level of NPT practice while on placement mandatory to encourage the development of NPT teaching skills that can translate to improved patient education skills as well as improved skills in communicating with colleagues.	
Educate key stake holders on benefits of NPT	
4. That in the process of providing training and pre-placement information to supervisors, RTPs and universities make the backgrounds of their registrars, PT and medical students known to supervisors, educate them on what juniors can bring to their practices and what type of teaching roles (and topics) may be appropriate for juniors depending on their level and backgrounds, and stress the importance of inviting them to teach.	
5. That RTPs and universities also educate learners on what they bring to the practice, which will improve their confidence to take on NPT roles.	
Educate supervisors	
6. That RTPs and universities provide feedback to supervisors on the importance of supporting learners with NPT teaching roles in the form of giving the learners tips, giving them feedback on how they went and how they could improve, and encouraging other learners to provide feedback also.	
7. That RTPs and universities provide information to supervisors on how to determine readiness to teach.	
Systems	
8. Increase exposure of all key stakeholders to NPT	
9. Develop systems for medical students to prepare resources for educational sessions for senior learners and GPs.	
10. NPT systems implementation should include a component that measures the learners’ level of knowledge in terms of postgraduate training, previous health-related careers, and basic sciences. This information should be used to develop local NPT training sessions.	
11. NPT practice implementation should coincide with developing compliance or quality assurance systems to ensure legal and professional rules and regulations are safeguarded.	
12. Reduce the reliance of senior registrars on fee for service and have them paid a salary via a central training body, as then taking on a clinical supervision load would not impact their income.	
Collaboration	
13. That RTPs and universities collaborate to provide opportunities for supervisors and multiple levels of learners to interact in a setting where learners can showcase their knowledge and skills.	

*RTP* regional training provider

Study limitations included reliance on the accuracy of self-report and the potential for socially desirable responding [38], however, the latter risk is lower in anonymous surveys [39]. Although this study involved a large national sample, those with a greater interest in shared learning may have been more likely to respond, as a much higher proportion of registrars reported doing some teaching than previously reported (24). Conversely, participants’ demographics were similar to national demographics [18] and the study included stakeholders that were not exposed to NPT and/or who had negative views towards it.

### Implications

To facilitate the process of implementing NPT, specific funding, policy initiatives, curriculum elements, and education systems need to be developed for NPT. A list of recommendations generated from the data is listed in Table 15. General practice training could learn from other specialties to fast track this process.

## Conclusions

This research offered an opportunity to examine the views of a national sample of key stakeholders in general practice in relation to NPT. While some NPT is occurring in general practice there is scope to increase it. Both supervisors and learners see benefits to NPT although they are more positive about the benefits to the learner-teacher than to the teaching recipients. Finding better structures to train, encourage and support learner-teachers may make NPT a more attractive option for learners and improve the quality of the teaching that they deliver.

### Availability of data and materials

Data can be obtained upon request from t.vandemortel@griffith.edu.au

## Abbreviations

NPT: near-peer teaching
SLL: single-level learner practice
MLL: multi-level learner practice
GPS: general practice supervisor
GPR: general practice registrar
PT: prevocational trainee
MS: medical student
RTP: regional training provider (of general practice training)
GPT1: general practice term 1
GPT2: general practice term 2
GPT3: general practice term 3
GPT4: extension of time term
ES: extended skills term
PGY1: postgraduate year 1
PGY2: postgraduate year 2
PGY3: postgraduate term 3
PIP: practice incentive payment
F: female

## Footnotes

1 Link to a copy of the supervisor and registrar surveys. Please note the survey was implemented in Survey Monkey, and therefore while it looks like the survey is long and some questions are repeated it used logic systems to direct survey participants through particular pathways in the questionnaire depending on their answers. For example, if the registrar answered that they did not do any teaching, they were not directed to the question that asked if they paid for that teaching or directed to questions asking what type of teaching they did.
